# Social and cultural features of cholera and shigellosis in peri-urban and rural communities of Zanzibar

**DOI:** 10.1186/1471-2334-10-339

**Published:** 2010-11-26

**Authors:** Christian Schaetti, Ahmed M Khatib, Said M Ali, Raymond Hutubessy, Claire-Lise Chaignat, Mitchell G Weiss

**Affiliations:** 1 Department of Epidemiology and Public Health, Swiss Tropical and Public Health Institute, Basel, Switzerland; 2 University of Basel, Basel, Switzerland; 3 Ministry of Health and Social Welfare of Zanzibar, Zanzibar, United Republic of Tanzania; 4 Public Health Laboratory Ivo de Carneri, Ministry of Health and Social Welfare of Zanzibar, Chake-Chake, Pemba, United Republic of Tanzania; 5 Initiative for Vaccine Research, World Health Organization, Geneva, Switzerland; 6 Global Task Force on Cholera Control, World Health Organization, Geneva, Switzerland

## Abstract

**Background:**

Responding to the high burden of cholera in developing countries, the WHO now considers vaccination as a supplement to the provision of safe drinking water and improved sanitation in the strategy for cholera control in endemic settings. Cultural concepts of illness affect many aspects of public health. In the first step of a two-step strategy to examine determinants of cholera vaccine acceptance, this study identified social and cultural features of diarrhoeal illness for cholera control in endemic communities.

**Methods:**

A cultural epidemiological study with locally adapted vignette-based interviews was conducted in two cholera-endemic communities of Zanzibar. A random sample of unaffected peri-urban (n = 179) and rural (n = 177) adults was interviewed to study community ideas of cholera and shigellosis, considering categories of distress, perceived causes, and help-seeking behaviour.

**Results:**

Cholera was recognised by 88%. Symptoms of dehydration were most prominent in reports at the peri-urban site. Interference with work leading to strain on household finances was frequently emphasised. Dirty environment was the most prominent perceived cause, followed by unsafe drinking water and germ-carrying flies. Causes unrelated to the biomedical basis of cholera were reported more often by rural respondents. Rural women had more difficulty (20%) to identify a cause than men (7.1%, p = 0.016). Peri-urban self treatment emphasised rehydration; the rural community preferred herbal treatment and antibiotics. Shigellosis was recognised by 70%. Fewer regarded it as very serious compared with cholera (76% vs. 97%, p < 0.001) and regarded it as less likely to be fatal (48% vs. 78%, p < 0.001). More respondents could not explain causes of shigellosis (23%) compared with cholera (7.3%, p < 0.001). Community respondents less frequently identified dehydration and contagiousness for shigellosis. Government facilities were preferred healthcare providers for both conditions.

**Conclusions:**

This study clarified local views of cholera and shigellosis relevant for diarrhoeal disease control in Zanzibar. The finding that rural women were less likely than men to specify causes of cholera suggests more attention to them is required. Better health education is needed for cholera in rural areas and for shigellosis in general. This study also identified variables for subsequent analysis of social and cultural determinants of cholera vaccine acceptance.

## Background

Cholera is an intestinal disease characterised by acute and profuse watery diarrhoea, caused by the bacterium *Vibrio cholerae *O1 or O139. A total of 190,130 cases and 5,143 deaths globally were reported to the World Health Organization (WHO) in 2008 [1], which is an underestimate; the annual burden is likely to exceed 3 million episodes and over 100,000 deaths [2,3]. The approach to control involves treatment of patients with rehydration and prevention of new cases, based on improved sanitation, hygiene and safe water supply. Because of persistence of cholera as a public health problem, the WHO now recommends vaccines as an additional tool to control cholera in endemic areas [3].

Cultural concepts about illness and how to treat and prevent it are important for many aspects of public health. The role of various social and cultural factors (e.g. socio-demographic characteristics, gender, urban and rural setting, and cultural concepts of illness and treatment) has practical implications for behaviour, public health, and disease control that need to be considered. Such factors are also likely to be especially important considerations for the acceptance and demand for vaccines [4-7]. Effective disease control with a vaccine requires not only an efficacious vaccine and health system to deliver it, but also recognition among the general population of its benefits and their willingness to use such a vaccine [8]. Consideration of cultural concepts of cholera and of a comparable serious disease, such as shigellosis, which has both similar and distinctive features, may help to formulate effective strategies, general and specific, for cholera control.

Studies have begun to address questions of vaccine acceptance and demand for diarrhoeal diseases, including recent research on typhoid fever and shigellosis in Asian countries [9-13], but not yet for cholera in Africa. Such research requires consideration of how cultural concepts of cholera affect acceptance and demand for a vaccine. To achieve that, two steps are essential: First, it is necessary to identify social and cultural features of the disease, and in a second step to explain how these features of cholera influence vaccine acceptance. This study was concerned with the first of these two questions, and the second will be addressed in a subsequent paper.

Fieldwork was undertaken in Zanzibar, motivated by the interest of the Ministry of Health and Social Welfare (MoHSW) in using a cholera vaccine for control in endemic peri-urban and rural areas of the archipelago. Because shigellosis, caused by enteropathogenic *Shigella *spp., is also endemic, and it has a profile of symptoms different from cholera, it was included for comparative study of local experience, meaning and preferred sources of help for diarrhoeal illness.

Specific aims of the study were (i) to examine the variety and distribution of social and cultural views of cholera, (ii) to compare these views in peri-urban and rural endemic communities, and (iii) to identify common and distinctive features of cholera and shigellosis that clarify how well differentiated these conditions are in these communities.

## Methods

### Setting and study sites

The survey was conducted from June to August 2008 in Zanzibar, United Republic of Tanzania. This Indian Ocean archipelago consists of two major islands – Unguja and Pemba – inhabited by a rapidly growing population of approximately 1.2 million Kiswahili-speaking people, who are predominantly Muslim. Medical morbidity in the population of Zanzibar mainly results from communicable diseases like upper respiratory tract infections, including pneumonia (33% of outpatient visits to primary and secondary hospitals in 2008), malaria (9.7%) and diarrhoeal diseases (8.6%) [14]. According to the latest Tanzanian national census (2002), the health situation on the islands has been improving, and the life expectancy at birth rose from 47 to 57 years between 1988 and 2002 [15].

A peri-urban and a rural community (locally termed Shehia) in core areas for a subsequent mass vaccination campaign were selected as study sites. This campaign with the killed whole-cell oral cholera vaccine Dukoral^® ^was conducted in January and February 2009. Interviews for this study were conducted simultaneously in the peri-urban Shehia of Chumbuni and the rural Shehia of Mwambe. A description of the study sites is given in Table 1. Both Shehias are served by a primary healthcare unit within walking distance, which is staffed with nurses and stocked with basic drugs and equipment mainly for outpatient treatment [16].

**Table 1:** Overview of study sites

	Peri-urban site	Rural site
**Administrative structure**		
Community (Shehia)	Chumbuni	Mwambe

District	Urban^a^	Mkoani

Island	Unguja	Pemba

**Population estimates**		

Number of inhabitants^b^	10,869	8,164

Population density	15,300/km^2^	800/km^2^

**Characteristics**		

Environment	Unplanned, slum-like extension of the capital, situated along a main road, narrow alleys, sandy ground, few trees and shrubs, few plots for farming	Coastline community with widely scattered hamlets, lush green vegetation, livestock, cassava, banana, paddy rice and coconuts

Main housing structure	Brick houses, corrugated iron roofs	Mud houses, thatched roofs

**Access to^c ^(%)**		

Electricity	34	6.2

Private or community piped water	84	59

Public wells	8.1	39

Latrines	70	32

No access to toilet facilities	7.0	57

**Economy^c^**		

Main economic activities	Informal business, government employees	Fishing, farming

Monthly median per capita expenditure	USD22.6	USD17.5

**Annual incidence of cholera per 1,000 population^d^**		

Mean (standard deviation)	2.9 (1.7)	2.5 (6.0)

Median (range)	2.2 (1.3-5.7)	0 (0-14.8)

**Annual incidence of shigellosis per 1,000 population^e^**		

Mean (standard deviation)	4.6 (1.6)	1.9 (0.3)

Median (range)	4.9 (2.9-6.1)	2.0 (1.7-2.2)

^a ^Despite belonging to the Urban district, this community is of peri-urban character; ^b^Census data from cholera control research project, 2008; ^c ^District-level data from Zanzibar Household Budget Survey, 2004/5 [16]; ^d ^Estimates (2002-2007) from Reyburn et al. (unpublished data) and WHO Cholera Country Profile for Zanzibar, 2006; ^e ^District-level estimates (2006-2008) from health facility-based surveillance [14,36]

### Research framework and instrument

Among the various formulations of cultural epidemiology for health social science research [17], this study is based on an approach for examining the distribution of community ideas of illness-related experience, meaning and behaviour [18,19]. A semi-structured Explanatory Model Interview Catalogue (EMIC) interview was developed to study community views of cholera and shigellosis in a peri-urban and rural community of Zanzibar. These EMIC interviews produce complementary data sets with numeric data for quantitative analysis and illness narrative data for qualitative analysis [20].

A first version of the interview was drafted in English during several scientific workshops and translated locally into Kiswahili. A series of focus group discussions and a field assistant training workshop with piloting of the instrument among people living adjacent to the study communities followed. This was crucial to further refine the EMIC interview with regard to clarity, field applicability and questions concerning translation. Because people without a current diarrhoeal disease were interviewed, rather than cases, the conditions that were the focus of the interview were introduced as clinical vignettes. For each condition, the respondent was asked to consider the case of a person typical of community residents with pathognomonic somatic symptoms presented in simple, easily understandable terms (see additional file 1). The sex of the vignette and respondent were matched. All questions of the interview that was based on the vignette referred to the diarrhoeal illness of the person described in the vignette.

Selected socio-demographic variables were recorded at the outset before enquiring about illness-related experience, meaning and behaviour operationalised as categories denoting patterns of distress (referring to additional somatic symptoms not mentioned in the vignettes and psycho-social problems), perceived causes, self treatment at home, and outside help seeking. The selection of the most relevant locally valid categories of distress, perceived causes, and help-seeking behaviour required for a meaningful description of the insider's perspective was based on discussions with local researchers, fieldworkers and focus group discussions among purposively selected community residents.

### Study design and participant selection

This cross-sectional survey was conducted prior to a mass oral cholera vaccination campaign to provide baseline data on community views of diarrhoeal illness in areas of Zanzibar at high risk for cholera among unaffected adults [21]. A simple random sample of 180 houses per site was drawn based on enumerated houses from an existing geographic information system for the peri-urban and a census database for the rural site. Sampled peri-urban houses were approached with the help of aerial photographs and a global positioning system device. Sampled houses in the rural community were located through census house numbers nailed on doorframes. If the house selected for sampling did not contain dwellings (e.g. if it was a business place, mosque or under construction), then the field teams would move on to the house which was closest to the front door of the originally selected house. If the second house was not inhabited either, then a third house was identified following the above procedure, and so forth until a household with eligible participants was found. A household is defined by people sharing the same kitchen or pot. Eligible participants had to be 18 years or older and willing enough to give time for an interview of approximately one hour duration.

Three field teams plus a coordinator on both islands were recruited by the MoHSW and trained in a ten-day workshop to conduct this survey. Each team consisting of an interviewer and a note taker completed on average two interviews per day. Written informed consent was obtained from all participants prior to the interview and no compensation was offered to them.

### Data management and analysis strategy

For cholera, the categories related to illness experience, meaning and help-seeking behaviour were coded for their prominence with a value of 2 after a spontaneous response, a value of 1 after a probed response and a value of 0 if not considered at all to reflect the response style. An additional value of 3 was assigned to the category of response if the category was considered the most troubling category of distress, the most important perceived cause or the most helpful self treatment or source of help. The cumulative prominence by respondent (ranging from 0-5) was then used to calculate the mean prominence for each category. Thematically similar individual categories were grouped under specific headings (e.g. related to dehydration among somatic symptoms) for the analysis of broader concepts of experience, meaning and behaviour. Calculation of the grouped prominence followed the same procedure as with the individual variables. To identify significant differences for cholera between the two sites and between sexes, a non-parametric statistic, the Wilcoxon rank-sum test, was used when comparing prominence variables; the Pearson Chi^2 ^and Fisher's exact test were applied when comparing proportions. This particular approach to comparing prominence, which has been widely used in other cultural epidemiological studies, takes more information about a category into account than a simple comparison of frequencies of report without considering how they are reported.

A similar series of questions were asked to elicit shigellosis-related illness experience, meaning and help-seeking behaviour. The same categories that were coded for cholera were also coded for shigellosis. Comparative analysis between the two conditions considered only spontaneously reported categories, because the interview coded only spontaneous responses for shigellosis. The proportion of positive responses by category was tabulated individually for each vignette, and for a report in both vignettes. To determine whether a category was associated more with one vignette than the other, McNemar's Chi^2 ^test for paired data was used. To examine whether or not individual categories were differentiated between both conditions, Cohen's kappa was calculated. The kappa statistic indicates the strength of agreement for a categorical assessment, corrected for agreement by chance. The analysis identified the two conditions as distinct for a category if the kappa coefficient was below 0.4, a level commonly accepted as a threshold for moderate agreement [22].

Narrative information was written down during the interview in Kiswahili, then translated into English and typed in a word processor. The qualitative software MAXQDA, version 2007, was used for managing the textual data and to facilitate further analyses of findings from quantitative data. Quantitative data was entered twice and verified in Epi Info software, version 3.4.3, and cleaned. Statistical analyses were done with Stata, version 10.

### Sample size

The sample size calculation was based on comparison of mean prominence of categories of distress, perceived causes, self treatment and outside help seeking for peri-urban–rural and female–male differences. The detection of a difference of 0.5 between prominence means with equal standard deviations of 1.5 at 95% significance and 80% power required a sample size of at least 164 individuals per independent group. This calculation was based on a two-sample t test assuming no underlying distribution in the data [23]. Ten percent was added to this sample size to compensate for missing data.

### Ethics

The protocol describing the study presented here was cleared by the WHO Research Ethics Review Committee and the MoHSW Ethics Committee in Zanzibar and later published in an open access journal to make it freely available to the research community [21]. Only individuals who gave written informed consent were interviewed. All data were handled with strict confidentiality and made anonymous before analysis.

## Results

### Sample characteristics

A total of 356 interviews were conducted, with very few people among the visited households who refused to be interviewed. The socio-demographic characteristics of the sample are summarised by site in Table 2. All respondents were Tanzanians and Muslims except a 22-year-old woman from Chumbuni who was Christian. The majority of the peri-urban sample consisted of married housewives and men doing small businesses. Peri-urban residents lived in bigger families than their rural counterparts and were also better educated. The rural sample in contrast consisted primarily of married persons mostly active in farming, fishing and also small informal businesses.

**Table 2:** Sample characteristics of study respondents from the general adult population of Zanzibar, n = 356

	Peri-urban site, n = 179	Rural site, n = 177
**Sex (%)**		

Female	48.6	52.0

**Age (years)**		

Mean (standard deviation)	36.5 (14.1)	34.4 (14.9)

Median (range)	35 (18-85)	30 (18-90)

Marital status^a ^** (%)		

Never married	23.5	11.9

Married	68.7	84.2

Separated	0.6	0.0

Divorced	4.5	3.4

Widowed	2.8	0.6

Household size^b ^*** (number of persons)		

Mean (standard deviation)	7.4 (3.2)	6.2 (2.7)

Occupation^a ^*** (%)		

Agriculture	4.5	57.1

Fishing	2.2	12.4

Self-employment	22.3	11.9

Formal employment	11.7	4.0

Housewife	33.5	9.0

Casual labourer	2.2	0.6

Student	14.5	4.0

Not active/retired	8.9	1.1

Highest educational level attained^c ^*** (%)		

No education	9.5	4.5

Koranic school	10.1	34.5

Primary school	23.5	33.9

Secondary school	54.2	25.4

Higher education	2.8	1.7

Education^c ^*** (years)		

Median (range)	10 (0-16)	6 (0-20)

**Household income (%)**		

More regular and dependable	59.8	52.0

Less regular and dependable	40.2	48.0

^a ^Pearson Chi^2 ^or Fisher's exact test; ^b ^t test; ^c ^Wilcoxon test

* p ≤ 0.05, ** p ≤ 0.01, *** p ≤ 0.001

### Recognition and importance of illnesses and past episodes

The vignette describing an adult person with symptoms of acute watery diarrhoea was named by 88.2% of the sample as *kipindupindu*, which is the Kiswahili name for the disease entity cholera. The rural villagers recognised cholera less often than the peri-urban residents (80.8% vs. 95.5%, p < 0.001, Chi^2 ^test). Other names given by rural villagers were *kuharisha kawaida *for normal diarrhoea (6.2%) and *kuharisha maji *for watery diarrhoea (4.0%) while 6.2% could not identify the condition at all. The condition described in the shigellosis vignette was identified by 69.9% of the respondents as *kuharisha damu*, which refers to the disease entity bloody diarrhoea. While 12.9% could not name it at all, 19 individuals (5.3%) confused the case presented in the shigellosis vignette with cholera.

The perceived severity and likely fatality for cholera and shigellosis vignettes was assessed in the peri-urban and rural areas. Cholera was more frequently said to be "very serious" (96.6%) than shigellosis (76.1%, p < 0.001, McNemar's Chi^2 ^test). Cholera was also more often anticipated to be "usually fatal without treatment" (77.5%) than shigellosis (47.8%, p < 0.001, McNemar's Chi^2 ^test). Although there was no difference in perceived severity for cholera at the two sites, for shigellosis more peri-urban respondents considered it very serious (86.0%) than rural respondents (66.1%, p < 0.001, Chi^2 ^test). Peri-urban respondents more frequently anticipated fatality for cholera (84.4%) than rural respondents (70.6%, p = 0.002, Chi^2 ^test), and peri-urban respondents were also more likely to anticipate fatality for shigellosis (65.4%) than rural respondents (29.9%, p < 0.001, Chi^2 ^test).

When asked about previous experiences of the condition described in the cholera vignette, 5.3% of the total sample reported an individual episode. Stratified analyses revealed a significant difference between the peri-urban and rural community (2.8% vs. 7.9%, p = 0.032, Chi^2 ^test), but not between women and men (3.4% vs. 7.3%, p = 0.094, Chi^2 ^test).

### Patterns of distress for cholera

Weakness was reported as the most prominent somatic symptom by the total sample (Table 3 upper panel). Categories related to dehydration, none of which were mentioned in the vignette, featured more prominently in the peri-urban site. This difference was primarily due to unconsciousness, a symptom which was identified by almost one-third of the peri-urban sample as most troubling. The respondents' views regarding this category were related to the loss of body fluid or the advanced stage of the illness. Almost one-fifth could not report any other somatic symptom apart from the ones described in the vignette. Symptoms related to shigellosis were probed for consistency under the cholera vignette but were less often mentioned spontaneously or identified as most troubling and hence yielded a lower prominence than symptoms of general gastroenteritis or dehydration.

**Table 3:** Somatic symptoms and psycho-social problems for a cholera vignette in peri-urban and rural Zanzibar, n = 356

	Peri-urban site, n = 179				Rural site, n = 177				
	How reported?^b^				How reported?^b^				
					
Category^a^	Total Reported %	**Fraction spon**.	Most troubling %	Mean prominence^c^	Total Reported %	**Fraction spon**.	Most troubling %	Mean prominence^c^	
*Somatic symptoms*									

**Related to general gastrointestinal illness**	**98.9**	**0.81**	**14.5**	**2.23**	**99.4**	**0.67**	**16.4**	**2.15**	

Abdominal pain/discomfort	91.1	0.47	2.8	1.42	88.7	0.06	6.2	1.13	***

Headache	64.8	0.02	0.0	0.66	55.4	0.02	0.6	0.58	

Loss of appetite	92.2	0.20	1.7	1.16	83.6	0.06	1.1	0.92	***

Nausea	87.7	0.04	0.6	0.93	88.1	0.03	0.6	0.93	

Weakness	96.6	0.69	9.5	1.92	97.7	0.64	7.9	1.84	

**Related to shigellosis**	**97.2**	**0.20**	**3.9**	**1.28**	**96.0**	**0.10**	**13.0**	**1.45**	

Abdominal cramps	76.5	0.11	2.8	0.93	75.1	0.08	6.8	1.01	

Bloody stool	23.5	0.14	0.6	0.28	50.3	0.03	2.8	0.60	***

Fever	82.7	0.11	0.6	0.93	87.0	0.03	1.7	0.95	

Pus in stool	8.9	0.06	0.0	0.09	37.9	0.00	0.0	0.38	***

Rectal pain	69.3	0.00	0.0	0.69	73.4	0.00	1.7	0.79	

**Related to dehydration**	**98.3**	**0.31**	**46.9**	**2.70**	**98.3**	**0.51**	**18.6**	**2.04**	**

Confusion	87.7	0.01	2.2	0.95	81.9	0.01	2.3	0.89	

Palpitations	84.4	0.03	9.5	1.16	73.4	0.02	1.7	0.80	***

Loose skin	90.5	0.17	1.1	1.09	88.1	0.24	0.6	1.11	

Sunken eyes	93.9	0.21	0.6	1.15	96.0	0.41	0.6	1.37	***

Unconsciousness	92.7	0.04	32.4	1.94	90.4	0.06	11.3	1.30	***

Very thirsty	76.5	0.03	1.1	0.82	78.5	0.06	2.3	0.90	

**Miscellaneous**	**25.1**	**1.00**	**1.7**	**0.55**	**38.4**	**1.00**	**1.7**	**0.82**	**

Other symptoms	10.1	1.00	1.1	0.23	16.4	1.00	0.6	0.34	

Cannot say	15.1	1.00	0.6	0.32	22.0	1.00	1.1	0.47	

*Psycho-social problems*									

**Social impact**	**99.4**	**0.88**	**36.9**	**2.97**	**99.4**	**0.91**	**50.3**	**3.41**	**

Disruption of health services	48.0	0.01	1.7	0.54	88.1	0.01	1.7	0.94	***

Fear of infecting others	83.8	0.28	2.2	1.14	72.9	0.22	6.8	1.09	

Fear of isolation from others	62.6	0.36	8.4	1.10	53.1	0.12	14.7	1.03	

Interference with social relationships	65.4	0.08	3.9	0.82	74.6	0.60	2.8	1.28	***

Interference with work/daily activities	96.6	0.73	20.7	2.30	97.2	0.72	24.3	2.40	

**Emotional impact**	**100.0**	**0.75**	**10.1**	**2.06**	**94.9**	**0.45**	**11.3**	**1.72**	***

Sadness, anxiety, worry	100.0	0.75	10.1	2.06	94.9	0.45	11.3	1.72	***

**Financial impact**	**99.4**	**0.62**	**52.5**	**3.18**	**99.4**	**0.73**	**38.4**	**2.88**	

Costs (transport, food, drugs)	97.2	0.08	34.1	2.07	96.0	0.32	13.6	1.67	

Loss of family income	98.3	0.58	18.4	2.11	92.7	0.52	24.9	2.16	

^a ^Categories ordered alphabetically within each group (bold). Categories reported by less than 5% not listed; ^b ^Columns indicate percentage of reported categories, fraction of spontaneously mentioned categories and whether a category was identified as most troubling; ^c ^Mean prominence based on values assigned to each reported category (0 = not reported, 1 = reported after probing, 2 = reported spontaneously, 3 = identified as most troubling).

Wilcoxon test used to compare mean prominence between both sites (* p ≤ 0.05, ** p ≤ 0.01, *** p ≤ 0.001).

When assessing the potential impact of cholera on a person's life, interference with work or daily activities was ranked as the highest category in both sites, followed by financial and emotional distress (Table 3 lower panel). The disruption of local health services was rated as the least important problem overall, but it was seen more as a problem in the rural community. The spontaneous account of a 75-year-old man from Chumbuni indicates how respondents describe the impact of cholera:

"It affects life in general. Emotionally, the patient thinks that he is going to die. Also, financially, he will spend a lot of money to buy medicine and at the same time he cannot work because of the disease."

The emotional impact was more prominently expressed in the peri-urban community, where the fraction of spontaneous replies for this category was higher. Despite this significant difference, the dangerousness of cholera, especially in relation to the possibility of death as exemplified in the statement above, featured equally in both communities.

### Perceived causes for cholera

A dirty environment (*mazingira machafu*), related to general in-and outdoor dirtiness, was by far the most prominently reported perceived cause overall, but particularly notable in the peri-urban site (Table 4). Among the causes related to ingestion, which were the second most prominent group in both sites, drinking contaminated water was ranked highest. This category was coded when respondents mentioned drinking unboiled or dirty water, or water containing faeces – some respondents explicitly mentioned cholera bacteria. Drinking contaminated water ranked as the second most prominent cause in total followed by flies, which were seen as disease transmitters in both communities. Flies, which can actually transmit *V. cholerae *[24,25], were mostly mentioned in connection with uncovered, i.e. unprotected, food, which was more prominently reported in the peri-urban community:

"Yes, because usually flies carry dirt and spread it everywhere, especially in the food." (Housewife from Chumbuni, 32 years old)

"It is possible that the flies coming from the toilet contaminate the food." (Male coffee seller from Mwambe, 50 years old)

**Table 4:** Perceived causes for a cholera vignette in peri-urban and rural Zanzibar, n = 356

	Peri-urban site, n = 179				Rural site, n = 177				
	How reported?^b^				How reported?^b^				
					
Category^a^	Total Reported %	**Fraction spon**.	Most important %	Mean prominence^c^	Total Reported %	**Fraction spon**.	Most important %	Mean prominence^c^	
**Ingestion**	**98.3**	**0.61**	**17.3**	**2.11**	**96.6**	**0.37**	**22.6**	**2.00**	*

Drinking contaminated water	96.1	0.40	10.1	1.65	94.4	0.24	16.4	1.66	

Eating unprotected/spoiled food	95.5	0.45	7.3	1.60	94.4	0.18	5.1	1.27	***

Eating forbidden food	27.4	0.00	0.0	0.27	54.8	0.00	1.1	0.58	***

Eating soil	36.9	0.00	0.0	0.37	48.6	0.01	0.0	0.49	*

**Behaviour**	**96.1**	**0.28**	**4.5**	**1.36**	**94.4**	**0.44**	**11.3**	**1.69**	**

Contact with contaminated water	85.5	0.20	1.7	1.07	91.0	0.42	9.6	1.58	***

Not washing hands	92.2	0.14	2.8	1.13	88.1	0.12	1.7	1.03	

**Environment**	**100.0**	**0.89**	**70.9**	**4.02**	**98.3**	**0.68**	**37.3**	**2.77**	***

Dirty environment	99.4	0.84	61.5	3.68	96.0	0.62	24.9	2.30	***

Flies	99.4	0.34	9.5	1.62	94.4	0.28	12.4	1.58	

Malaria	15.1	0.00	0.0	0.15	48.0	0.02	0.0	0.49	***

Worms	13.4	0.00	0.0	0.13	46.9	0.00	0.0	0.47	***

**Magico-religious causes**	**94.4**	**0.07**	**7.3**	**1.23**	**91.0**	**0.16**	**28.8**	**1.92**	***

God's will	93.3	0.07	7.3	1.22	86.4	0.16	27.7	1.83	***

Witchcraft	20.7	0.00	0.0	0.21	45.8	0.01	1.1	0.50	***

**Miscellaneous**	**5.0**	**1.00**	**0.0**	**0.10**	**27.1**	**1.00**	**0.0**	**0.54**	***

Other	3.9	1.00	0.0	0.08	13.6	1.00	0.0	0.27	**

Cannot say	1.1	1.00	0.0	0.02	13.6	1.00	0.0	0.27	***

^a ^Categories ordered alphabetically within each group (bold), except "cannot say". Categories reported by less than 5% not listed; ^b ^Columns indicate percentage of reported categories, fraction of spontaneously mentioned categories and whether a category was identified as most important; ^c ^Mean prominence based on values assigned to each reported category (0 = not reported, 1 = reported after probing, 2 = reported spontaneously, 3 = identified as most important).

Wilcoxon test used to compare mean prominence between both sites (* p ≤ 0.05, **p ≤ 0.01, *** p ≤ 0.001).

Among the causes not related to the faecal-oral route of transmission, God's will was the most prominent category and ranking higher among rural residents. A statement from a 30-year-old female farmer from Mwambe helps to explain the commonly expressed notion regarding this finding, i.e. that God overrules people's prevention efforts if only it wished:

"There is no cause except God's will, which cannot be changed; and it is not caused by dirty environment because there are some dirty places where people do not get the disease."

Further perceived causes not linked to cholera disease aetiology – like witchcraft, malaria and worms – had lower prominence ratings since they were almost never mentioned spontaneously nor identified as most important. And these categories were more characteristic for the rural compared with the peri-urban community. A substantial proportion of the respondents from Mwambe – more than one-tenth, compared to only two peri-urban residents – had no idea what could have made the person suffer from the symptoms described in the vignette (coded as cannot say). Among rural respondents who could not spontaneously identify a cause, women featured significantly more often than men (19.6% vs. 7.1%, p = 0.016, Wilcoxon test) (not shown in Table 4).

### Self treatment and help seeking for cholera

The most prominent self treatment at home in the rural community was herbal treatment, followed by giving antibiotics or other drugs like pain killers or antacids and then home-made or ready-to-use oral rehydration solution (ORS) (Table 5 upper panel). In contrast, the peri-urban residents' preference for herbal treatment was less pronounced as they primarily suggested giving someone like the person described in the cholera vignette more water or other liquids, like tea or porridge, or ORS. For most respondents, local herbal treatment, used for relief or cure of symptoms, comprised concoctions of water with locally grown spices like cumin or cloves, or with leaves, barks and roots of herbs and trees (e.g. *mpatakuva *(*Plectranthus *spp.), neem tree, guava). Doing nothing at home, i.e. sending the person described in the cholera vignette immediately to allopathic healthcare facilities, was considered as the least prominent category in the rural community, while it ranked fourth in the peri-urban community and was regarded as the most helpful thing one can do at home. The following statement from a housewife, aged 47 years, from Chumbuni is typical for what the communities would do for people with cholera at home:

"At home we give water and other people give local treatment. [...] and if the condition becomes worse, we will send the patient to the hospital."

**Table 5:** Self treatment and help seeking for a cholera vignette in peri-urban and rural Zanzibar, n = 356

	Peri-urban site, n = 179				Rural site, n = 177				
	How reported?^b^				How reported?^b^				
					
Category^a^	Total reported %	**Fraction spon**.	Most helpful %	Mean prominence^c^	Total reported %	**Fraction spon**.	Most helpful %	Mean prominence^c^	
*Self treatment at home*									

Antibiotics/drugs	44.7	0.26	15.6	1.03	72.3	0.14	24.9	1.57	***

Doing nothing at home	27.9	1.00	22.9	1.25	19.8	1.00	4.5	0.53	**

Drinking more water or liquids	68.7	0.45	19.6	1.58	69.5	0.10	9.6	1.05	**

Herbal treatment	49.7	0.75	14.0	1.29	83.1	0.73	28.8	2.31	***

Oral rehydration therapy/solution	59.8	0.28	21.2	1.40	72.9	0.07	23.7	1.49	

Prayers	55.9	0.02	5.6	0.74	47.5	0.00	8.5	0.73	

*Outside help seeking*									

Faith healers	11.7	0.00	0.0	0.12	18.1	0.00	2.3	0.25	

Health facilities	100.0	1.00	95.5	4.87	100.0	1.00	80.2	4.41	***

Informal help from health worker/friend	38.5	0.00	4.5	0.52	73.4	0.00	15.8	1.21	***

Pharmacy/OTC	27.4	0.00	0.0	0.27	40.7	0.00	1.1	0.44	**

Traditional healers	3.9	0.00	0.0	0.04	9.6	0.06	0.6	0.12	*

^a ^Categories ordered alphabetically. Categories reported by less than 5% not listed; ^b ^Columns indicate percentage of reported categories, fraction of spontaneously mentioned categories and whether a category was identified as most helpful; ^c ^Mean prominence based on values assigned to each reported category (0 = not reported, 1 = reported after probing, 2 = reported spontaneously, 3 = identified as most helpful).

Wilcoxon test used to compare mean prominence between both sites (* p ≤ 0.05, ** p ≤ 0.01, *** p ≤ 0.001).

Public primary healthcare units and hospitals were mentioned by all respondents (Table 5 lower panel). More than 95% of the peri-urban residents identified health facilities as most helpful source of treatment, while the rural residents' preference was around 15% lower. Faith healers and traditional healers were of little importance and probing revealed that they would only be consulted after allopathic treatment had failed.

### Shigellosis versus cholera

Similar to the cholera vignette, weakness was also rated as the most prominent somatic symptom for the shigellosis vignette (Table 6 top panel). Among symptoms related to dehydration, only loose skin and sunken eyes were mentioned; and both categories were reported significantly less for shigellosis than for cholera. The remaining symptoms of dehydration fell under the 5% threshold. All categories of somatic symptoms were differentiated on the individual level in both sites.

**Table 6:** Symptoms, perceived causes, and self treatment for a cholera and a shigellosis vignette in Zanzibar, n = 356

	Only cholera vignette^b^	Only shigellosis vignette^b^	Both cholera & shigellosis vignette^c^		Kappa coefficient^e^	
					
Category^a^	%	%	%	p value^d^	Estimate	95% CI
*Somatic symptoms*						

Abdominal pain/discomfort	24.2	38.2	13.2	**< 0.001**	0.18	0.08 - 0.28

Loose skin	18.3	5.6	3.4	**< 0.001**	0.21	0.09 - 0.34

Loss of appetite	11.8	13.5	3.1	0.467	0.14	0.01 - 0.26

Sunken eyes	29.5	8.4	5.1	**< 0.001**	0.16	0.06 - 0.25

Weakness	64.9	57.0	45.2	**0.008**	0.34	0.25 - 0.44

Other somatic symptoms	13.2	12.4	2.5	0.726	0.08	-0.04 - 0.20

Cannot say	18.5	22.8	9.8	0.087	0.34	0.23 - 0.46

*Psycho-social problems*						

Costs (transport, food, drugs)	19.1	20.8	12.6	0.405	**0.54**	0.43 - 0.65

Fear of infecting others	19.7	2.0	1.1	**< 0.001**	0.07	-0.01 - 0.15

Fear of isolation from others	14.3	4.2	2.0	**< 0.001**	0.16	0.03 - 0.29

Interference with social relationships	24.7	15.2	12.6	**< 0.001**	**0.55**	0.44 - 0.65

Interference with work/daily activities	70.5	61.8	53.9	**0.001**	**0.46**	0.35 - 0.55

Loss of family income	52.8	43.5	34.0	**0.001**	**0.44**	0.35 - 0.53

Sadness, anxiety, worry	59.3	58.7	47.2	0.827	**0.51**	0.42 - 0.60

*Perceived causes*						

Contact with contaminated water	27.5	2.8	1.7	**< 0.001**	0.06	-0.01 - 0.13

Dirty environment	71.6	32.6	28.4	**< 0.001**	0.17	0.11 - 0.25

Drinking contaminated water	30.6	21.6	9.6	**0.003**	0.15	0.04 - 0.26

Eating unprotected/spoiled food	30.1	29.8	12.1	0.929	0.15	0.04 - 0.26

Flies	30.3	13.2	5.9	**< 0.001**	0.11	0.01 - 0.21

God's will	10.1	13.8	3.9	0.085	0.24	0.10 - 0.38

Not washing hands	11.5	9.0	1.1	0.264	0.01	-0.10 - 0.12

Cannot say	7.3	23.0	5.6	**< 0.001**	0.29	0.18 - 0.41

*Self treatment at home*						

Antibiotics/drugs	11.0	20.5	3.4	**< 0.001**	0.08	-0.03 - 0.19

Doing nothing at home	23.9	20.2	10.4	0.154	0.32	0.21 - 0.44

Drinking more water or liquids	18.8	10.7	5.9	**< 0.001**	0.31	0.18 - 0.43

Herbal treatment	49.2	55.6	35.7	**0.035**	0.33	0.24 - 0.43

Oral rehydration therapy	11.0	5.9	2.8	**0.004**	0.28	0.12 - 0.44

^a ^Categories ordered alphabetically, except "cannot say". Categories reported by less than 5% of the sample for each vignette not listed; ^b ^Proportion of categories reported spontaneously for either cholera or shigellosis vignette; ^c ^Proportion of categories reported spontaneously for both vignettes; ^d ^McNemar's Chi^2 ^test used to compare population proportions between both vignettes. Bold figures (p ≤ 0.05) indicate significant differences; ^e ^Kappa coefficients (presented with 95% confidence intervals) greater than or equal to 0.4 suggest no differentiation of illness categories (bold figures).

Notable among psycho-social problems was fear of infection and fear of isolation from others. Both categories were reported considerably less for shigellosis than for cholera, and were also well-differentiated (Table 6 second panel). All the other categories, which represent general features of diarrhoeal illness, i.e. costs, loss of family income, interference with social relationships and with daily activities, and being sad, anxious or worried, were not differentiated between both conditions (kappa coefficient greater than 0.4).

A dirty environment was perceived to be the most prominent cause of shigellosis (Table 6 third panel). The percentage of this category, however, was less than half the percentage for cholera, and was closely followed by the category of eating unprotected or spoiled food. All categories of perceived causes that showed a significant difference between the two conditions were mentioned less frequently for shigellosis, with the exception of cannot say, which was reported three times more often for shigellosis than for cholera. Kappa coefficients for all categories were below the threshold of 0.4 suggesting differentiation of the meaning of cholera from shigellosis.

The distribution of respondents' answers for self-treatment options showed that the population proportions related to rehydration were higher for cholera (Table 6 bottom panel). Conversely, a likely benefit for shigellosis was reported for antibiotics/drugs and for herbal treatment. Kappa coefficients were also below the threshold of 0.4 for all help-seeking categories.

Similar to the observed preponderance in the case of cholera, health facilities were regarded as the sole source of outside help for treating people with shigellosis (354 out of 356 respondents).

## Discussion

Findings from both peri-urban and rural areas of Zanzibar were notable for the high perceived severity and anticipated fatality of cholera. Even though the condition described in the cholera vignette was similarly regarded as very serious in both communities, it was more often named as cholera and considered as a serious life-threatening illness in the peri-urban community. The lower recognition of the condition described in the cholera vignette in the rural community, which is consistent with lower prominence of reported signs and symptoms of dehydration and higher prominence for the two most conspicuous shigellosis signs (bloody stool, pus in stool), may be explained by poorer education. It cannot be explained by less personal illness experience of cholera, however, since rural residents reported the occurrence of an individual episode 2.8 times more often than peri-urban residents.

The severity of the condition in the cholera vignette was also elaborated with reference to its impact on affected persons and household livelihoods. Absence from work was felt to be the major effect at both sites leading to strain for household finances because of reduced or lost income and treatment costs. Compared to the shigellosis vignette, the condition described in the cholera vignette was more often perceived as a severe and potentially fatal health problem in both communities. This finding is consistent with another study comparing the two conditions; unaffected community residents, confirmed shigellosis patients and healthcare providers in Bangladesh considered cholera to be more severe than shigellosis [26].

Although a variety of causes were acknowledged, respondents clearly regarded the condition depicted in the cholera vignette as a disease linked to a dirty environment and to ingesting microbiologically contaminated water and food. The relevance of this concept of dirtiness and of sanitation and hygiene in connection with diarrhoea was also found in a qualitative study of childhood diarrhoea among mothers living in Chake-Chake district on Pemba [27]. The role of a dirty environment as a cause of cholera was especially highlighted by peri-urban residents living in an area with better water supply and sanitation. While it can be expected that better water supply and sanitation would result in less importance of dirty environment, the peri-urban emphasis in this study may be explained by the 19 times higher population density and the higher number of persons living in the average Chumbuni household. Most people reported magico-religious causes, but the relative priority was higher in the rural site. Other causes unrelated to the biomedical basis of cholera (i.e. worms and malaria) were less frequently mentioned in both sites and were also more prominent in the rural site. These findings are consistent with the lack of knowledge of cholera causes in the rural community, which was especially prominent among the women there.

Besides using various allopathic and traditional home remedies, respondents also recommended immediate hospital treatment when queried about what they would do at home with someone like the person described in the cholera vignette. Peri-urban community responses emphasised rehydration; rural community responses emphasised herbal treatment and use of antibiotics and other drugs. Certain herbs and plants, most of which were also reported as herbal treatment in the childhood diarrhoea study from Chake-Chake [27], were frequently recommended as home-based treatment. Reasons for that may include their availability to people, who collect them freely in the bush and woods, and their beneficial effect against cholera and other bacterial gastrointestinal diseases [28-30]. Peri-urban recommendations for self-treatment more frequently referred to health education and awareness, which probably results from exposure to public health activities. Peri-urban respondents also more frequently considered the value of immediate hospital treatment for the condition in the cholera vignette. Rural respondents, on the other hand, emphasised magico-religious and other unrelated causes of cholera.

In both sites, help seeking outside the household for the person described in the cholera vignette essentially meant going to public healthcare facilities, with little mention of traditional healers and faith healers. This finding of reliance on hospital treatment is remarkable compared with other studies from low-and middle-income countries, which emphasise traditional treatment for childhood and adult diarrhoea [31-33]. Several factors may help explain this priority: Many people in these communities have experience and a high regard for cholera treatment camps, which have been established when needed for outbreaks by the district administration and provide free treatment. Traditional health care providers, on the other hand, charge for their services. These communities have also been exposed to health education from public health action of the MoHSW and international non-governmental organisations in the wake of cholera outbreaks. Ethnographic field study also indicates that traditional healers in the study communities support hospital treatment (A. Pach, unpublished data).

The analysis of disagreement showed illness concepts for the two conditions were distinct with respect to reported patterns of distress, perceived causes and self treatment. For outside help seeking, however, reference to the value of hospital treatment was the same for both conditions. Differentiation of the two conditions may be explained by community and personal experience with cholera and shigellosis, resulting in the awareness of particular features of the two conditions. Both conditions occur with similar rates in the study communities (Table 1).

Health educational activities for cholera, in response to the priority arising from outbreaks making heavy demands on the health system in Zanzibar, are more extensive than for shigellosis. Less emphasis on shigellosis control may account for the finding that fewer respondents could explain the cause of shigellosis (23% reporting cannot say) compared with cholera (7.3%). The finding that fewer respondents identified houseflies as a cause of shigellosis may also result from the lower priority of public health action for shigellosis control, inasmuch as houseflies are recognised agents of transmission for shigellosis [34]. Dehydration and contagiousness are two other features of both conditions that community respondents identified more with cholera only. Dehydration is also an important feature of shigellosis, and shigellosis is more contagious than cholera [35].

The differentiation of the two conditions is reflected by appropriate differences in treatment recommended by respondents. Community self-treatment priorities emphasised rehydration for cholera and herbal and antibiotic treatment for shigellosis.

### Strengths and limitations

This study shows how EMIC interviews can be used to assess explanatory models of diarrhoeal illnesses among unaffected community residents and how to compare them among sub-groups. The specific approach employed in this cultural epidemiological study to comparing prominence allowed the ranking of categories according to their relative priority and not just according to their reported frequency. This weighted approach represents a more sensitive method to clarify differences between groups and has implications for explaining cultural priorities and potential effects on health behaviour.

The findings presented here are specific for cholera and shigellosis in one culture and focus on variation between peri-urban and rural areas. Thus, any generalisations made to countries outside the target populations have to be examined cautiously as the results presented here are inherently linked to the context. Some may argue that the differences in community views are due to education rather than to residence. However, analysis of the patterns of distress, perceived causes, self treatment and help seeking, stratified by educational status, showed that the cross-site differences reported here were not confounded by education.

It should also be noted that findings reported here are cross-sectional and may change over time, possibly in response to access to health services, a vaccine campaign or other social changes. Furthermore, the data are based on respondents' ideas about the condition of a clinical vignette, representing community views of illness experience, meaning and behaviour, but not necessarily an account of personal or family history.

The sampling included only community residents who were at home when the field teams visited. The study could be biased if the views of the respondents available for interviews at home and persons unavailable because of other responsibilities differed. The age distribution at both sites, however, mitigates this concern, inasmuch as all age groups were represented in the sample.

## Conclusions

This study has clarified local peri-urban and rural views of cholera among the general population with practical significance for cholera control in Zanzibar. Cholera was recognised as a serious and potentially fatal condition, a priority that makes such communities receptive to community health education programmes. The overwhelming preference for public healthcare facilities to treat cholera and shigellosis indicates the importance of strengthening health systems to ensure they are capable of fulfilling expectations. Notwithstanding this appropriate community preference for hospital treatment, this study also suggests that better health education is needed for cholera in rural areas and for shigellosis in general. The finding that rural women were more likely than men to be unable to specify a cause of cholera indicates the need to ensure a gender-sensitive approach to control.

Although sanitation, hygiene and safe water are critical issues for diarrhoeal disease control, recent consideration of vaccines in endemic areas suggest an appealing complementary intervention. It is an approach that has been of considerable interest to policy makers in Zanzibar, where a cholera vaccine campaign was implemented in January and February 2009. Research is needed to identify not only health system capacities to deliver vaccines but also social and cultural factors affecting community acceptance of vaccines. Factors influencing the willingness and enthusiasm of communities for a recommended vaccine can be expected to affect the success of a vaccine intervention programme. The interests and findings of this study are likely to inform such efforts to clarify social and cultural features of vaccine acceptance and demand.

Although not used in planning the cholera vaccine campaign in Zanzibar, findings from this study identified variables for a subsequent analysis of social and cultural determinants of vaccine acceptance and demand. Further analysis is also needed to explain the impact of the vaccine campaign on community views of cholera and risk-related behaviour. This study indicates directions and enables further research, and it has also clarified important issues for cholera control.

## Competing interests

The authors declare that they have no competing interests.

## Authors' contributions

Conception and design of this study: CS, AMK, SMA, RH, CLC and MGW. Supervision of data collection and data entry: CS. Analysis of data: CS and MGW. Writing of manuscript: CS and MGW. Revision and final approval of manuscript: CS, AMK, SMA, RH, CLC and MGW.

## Pre-publication history

The pre-publication history for this paper can be accessed here:

http://www.biomedcentral.com/1471-2334/10/339/prepub

## Supplementary Material

Additional file 1 Clinical vignettes for community study of cholera and shigellosis. Click here for file
