# Supplementary material for: Social and cultural features of cholera and shigellosis in peri-urban and rural communities of Zanzibar
Source: BMC Infect Dis. 2010 Nov 26;10:339. doi: 10.1186/1471-2334-10-339 (PMC3009639; doi:10.1186/1471-2334-10-339)
Supplement: Additional file 1 — Clinical vignettes for community study of cholera and shigellosis. [file 1471-2334-10-339-S1.PDF]

Clinical vignettes for community study of cholera and shigellosis

Cholera

[Name][1] from [community][2] who is 40 years old did not feel like going to visit his friends one morning last week. All of a sudden he had to run to the latrine. He became more and more concerned after the second and third time of running to the latrine that morning because he was passing lots of stool which looked like rice water. It was as if he were urinating instead of defecating. He also felt very miserable because he was vomiting terribly and the muscles in his arms and legs were very painful.

Shigellosis

When 25-year-old [name][1] woke up on Monday last week in his house in [community][2] he was feeling feverish and also had pangs of pain in his belly. He was having a loose stool and when he looked at it he noticed red drops, probably blood, and a whitish substance like pus. He went to the toilet another 3 times that day but he did not feel better afterwards. The day after, he still felt the same urge to go to the toilet many times. But with each time he produced less and less stool although he strained a lot and his anus was painful.

---

[1] Local names were used to make the story sound more familiar to the respondents. The names were always adapted to match the respondent's sex.

[2] *Chumbuni* was the community name for the peri-urban site on Unguja island and *Mwambe* for the rural site on Pemba island.
